# Psychological status and clinical outcomes in ankle arthrodesis: preoperative predictors and postoperative changes

**DOI:** 10.3389/fpsyt.2026.1691593

**Published:** 2026-02-13

**Authors:** ZhanHua Zhang, ShiHang Cao, Yi Li, Jun Lu, Dong Hu, JunKui Xu

**Affiliations:** Honghui Hospital, Xi’an Jiaotong University, Xi’an, Shaanxi, China

**Keywords:** ankle arthrodesis, ankle osteoarthritis, anxiety, depression, prognosis

## Abstract

**Purpose:**

The aim of this study is to investigate the psychological status of patients with end-stage ankle osteoarthritis (OA), and to evaluate the impact of ankle arthrodesis (AA) on the patients’ psychological condition, as well as the influence of preoperative anxiety and depression symptoms on the clinical outcomes after AA surgery.

**Methods:**

A retrospective observational study was conducted on 62 patients with end-stage ankle OA who underwent AA treatment at our hospital from January 2019 to December 2024. Based on the patients’ preoperative psychological status, they were divided into two groups: those with symptoms of anxiety/depression were included in Group A, and those without symptoms of anxiety/depression were included in Group B. The Hospital Anxiety and Depression Scale (HADS), the American Orthopaedic Foot and Ankle Society (AOFAS) score, and the Visual Analogue Scale (VAS) for pain were used to evaluate the patients before surgery and at the final follow-up. Independent sample t-tests and chi-square tests were used for between-group comparisons, and paired sample t-tests were used for within-group pre-post comparisons. P < 0.05 was considered statistically significant.

**Results:**

Of the 62 patients with end-stage ankle OA who were fully followed up, 30 had symptoms of anxiety/depression before surgery, a prevalence rate of up to 48%. All evaluation indicators for patients in Groups A and B improved significantly after AA surgery compared to before surgery, but the overall prognosis for Group A was worse than Group B.

**Conclusion:**

AA can effectively improve patients’ pain, functional activity, and psychological condition, and there is a significant correlation between the patients’ preoperative psychological status and the prognosis.

## Introduction

Ankle osteoarthritis (OA), as a chronic progressive disease, affects approximately 1% of the global population (1, 2). While the prevalence of ankle OA remains comparatively lower than that observed in hip or knee OA (3, 4), it nonetheless substantially impairs patient quality of life through persistent pain and functional limitations, with severe cases potentially progressing to disability (5). Moreover, unlike hip or knee OA, trauma rather than primary degeneration constitutes the predominant etiology of ankle OA (6, 7). Therefore, patients with ankle OA are younger compared to those with other degenerative joint diseases of the lower limbs (1), and they may require a higher level of functional activity.

Studies have reported that regardless of the stage of ankle OA progression, conservative treatment should be attempted for at least 6 months to evaluate therapeutic response (3). The majority of patients with early-stage ankle OA achieve satisfactory outcomes through non-operative interventions (5, 8, 9). However, in the late stages, conservative treatment is often minimally effective, and ankle arthrodesis (AA) or total ankle arthroplasty (TAA) may be necessary (10, 11). Veljkovic et al. demonstrated that patients receiving AA or TAA exhibited comparable clinical outcomes postoperatively (12). However, due to the high revision rate, high reoperation rate, and long learning curve associated with TAA, AA remains the most common surgical approach for treating end-stage ankle OA (3, 13–15).

Patients with end-stage ankle OA experiencing prolonged pain and persistent symptoms may be susceptible to developing psychological disturbances. Furthermore, individuals with compromised psychological well-being often exhibit heightened sensitivity to pain perception and symptom severity (16, 17). Prior investigations have indicated that preoperative psychological status may function as a prognostic indicator for clinical outcomes following ankle orthopedic procedures (18). Accordingly, addressing and optimizing patients’ psychological well-being prior to surgery may contribute to enhanced therapeutic outcomes. The objective of this study is not only to investigate the preoperative psychological status of patients with end-stage ankle OA and evaluate the impact of psychological status on the effectiveness of AA but also to elucidate the influence of AA on the psychological status of patients, aiming to develop personalized treatment plans for patients.

## Patients and methods

### Patients

We conducted a retrospective observational study of patients who underwent AA treatment in the Foot and Ankle Surgery Department of Xi’an Honghui Hospital from January 2019 to December 2024. We retrospectively identified all patients who had completed ankle arthrodesis during this period and then applied the following selection criteria. The inclusion criteria were as follows: (1) patients with end-stage ankle OA (Kellgren and Lawrence grade 4) who had persistent symptoms after conservative treatment (19); (2) patients without a history of contralateral ankle arthrodesis surgery; (3) patients with a minimum follow-up period of 1 year after surgery; (4) patients with an American Society of Anesthesiologists (ASA) physical status classification of grade 1-2. The exclusion criteria were as follows: (1) patients with severe osteoporosis, ankle joint infection, and other conditions; (2) patients with chronic internal medicine diseases such as diabetes and malignant tumors; (3) patients with psychiatric disorders other than anxiety/depression.

### Methods

Patients were evaluated preoperatively and at the final follow-up using the American Orthopaedic Foot and Ankle Society (AOFAS) ankle and hindfoot score, visual analogue scale (VAS) for pain assessment, and Hospital Anxiety and Depression Scale (HADS). We contacted patients through the WeChat (Tencent, Shenzhen, China) internet platform or by phone, and completed the questionnaire survey through face-to-face communication with the patients. The total score of the AOFAS scoring system is 100 points, which combines subjective and objective data to provide a comprehensive functional assessment of the ankle joint in patients (20). The VAS scoring scale has a range of 0–100 mm, with higher scores indicating more severe pain intensity (21). We used the HADS scoring scale to assess the psychological status of patients preoperatively and at the final follow-up. The HADS consists of 14 items, with 7 items assessing anxiety (A, 0–21 points) and 7 items assessing depression (D, 0–21 points). A score of 8 or higher indicates the presence of possible anxiety/depression symptoms (22).

We divided the patients into two groups based on their preoperative psychological condition. Group A consisted of patients with preoperative symptoms of anxiety/depression, and Group B consisted of patients without preoperative symptoms of anxiety/depression. All patients underwent surgery performed by the same experienced and senior orthopedic foot and ankle surgeon. Postoperatively, patients received identical rehabilitation training guidance.

### Surgical techniques and rehabilitation plan

All patients underwent the same anesthesia and surgical techniques, performed by the same group of doctors. 0.5% ropivacaine was used for sciatic nerve and femoral nerve block. After confirming the effectiveness of nerve block, general anesthesia was induced, and the surgery commenced immediately. Expose the ankle joint, and under direct visualization, use an oscillating saw and bone knife to remove the articular cartilage and necrotic bone from the distal tibial joint surface and the joint surface of the proximal talus. Fresh blood is visible under direct visualization, and after irrigation, remove bone debris. Perform drilling of the tibia and talus using Kirschner wires, along with micro-fracture surgery. Use a guide pin to guide the hollow nail and fix the tibio-talus joint at a 90-degree angle to the ankle joint, with approximately 5 degrees of eversion and external rotation. Intraoperatively, under fluoroscopy with a C-arm machine, observe a well-fused ankle joint with secure internal fixation. Remove the hollow nail guide pin, irrigate, and suture the incision.

Postoperatively, a brace was used to immobilize the affected ankle, the limb was elevated and appropriately iced to control swelling, and weight-bearing or dangling of the limb was avoided. One week postoperatively, encourage and guide the patient to perform lower limb muscle training and toe exercises. Starting from the first month postoperatively, instruct the patient in midfoot and hindfoot mobility exercises. At two months postoperatively, encourage weight-bearing walking with a walking boot. By the third month postoperatively, simple physical activities can be resumed, guiding the patient through full weight-bearing resistance training in a standing position. Regular follow-up examinations are necessary to observe bone healing progress.

### Statistical analysis

#### Software and data distribution testing

Statistical analyses were performed using SPSS 25.0 software (IBM, New York, United States). The Shapiro-Wilk test was used to assess the normality of all continuous variables. Descriptive statistics were reported as mean ± standard deviation (x¯ ± s) for normally distributed data.

#### Baseline comparisons and homogeneity testing

Independent sample t-tests were used to compare continuous baseline characteristics (age, follow-up time, duration of illness) between Groups A and B. Chi-square tests were employed for categorical variables (sex) to ensure homogeneity between groups.

### Within-group analyses

Paired sample t-tests were conducted to analyze the changes between preoperative and postoperative evaluation indicators (VAS, AOFAS, HADS-A, and HADS-D scores) within each group.

### Between-group analyses

Independent sample t-tests were used to: (1) compare preoperative evaluation indicators between the two groups; (2) compare postoperative evaluation indicators between the two groups; and (3) assess the differences in the degree of improvement of evaluation indicators between the two groups postoperatively.

### Correlation analyses for Group A

For Group A patients, Pearson correlation analysis was used to evaluate the relationship between continuous variables (age, duration of illness) and the degree of anxiety/depression. Independent sample t-tests were used to assess the association between sex and psychological status. The relationships between preoperative psychological scores and postoperative improvement were also examined using Pearson correlation analysis.

### Statistical significance

A significance level of P < 0.05 was considered statistically significant for all analyses.

### Ethics approval and consent to participate

The study was conducted in accordance with the ethical standards outlined in the Declaration of Helsinki. This study obtained approval from the Ethics Review Committee of Xi’an Honghui Hospital (NO.202401028). Prior to inclusion in the study, informed consent was obtained from all patients.

## Results

### General condition of the patient

A total of 67 patients who met the criteria of this study were followed up, with 62 patients completing the entire follow-up, including 23 males and 39 females. The mean age was 60.52 ± 8.88 years, with an average duration of illness of 10.56 ± 7.73 years, and an average follow-up time of 29.32 ± 10.54 months. 30 patients had anxiety/depression symptoms before surgery, accounting for 48%, including 12 males and 18 females. 32 patients had no anxiety/depression symptoms before surgery, accounting for 52%, including 11 males and 21 females. The baseline characteristics of the two groups of patients are shown in **Table 1**. There were no statistically significant differences between the two groups of patients in terms of age, gender, duration of illness, and follow-up time, as shown in **Table 1**.

**Table 1:** General characteristics of the patients.

Characteristics	Group A (n=30)	Group B (n=32)	P value
Sex, n (%)			
Male	12(40%)	11(34%)	0.647
Female	18(60%)	21(66%)	
Age (years)	59.40 ± 8.89	61.56 ± 8.88	0.342
follow-up time (months)	29.63 ± 10.25	29.03 ± 10.96	0.824
Duration of illness (years)	9.56 ± 6.33	11.50 ± 8.84	0.324
HADS-A (preop)	9.27 ± 1.36	5.06 ± 1.11	<0.001
HADS-D (preop)	8.87 ± 1.38	4.94 ± 1.46	<0.001

### Efficacy analysis

During the follow-up process, we did not observe any complications such as infection, abnormal healing, delayed healing, or nonunion in the patients. At the final follow-up, patients in Group A showed significant improvements in psychological status, pain level, and overall ankle joint function compared to before surgery (P<0.001, **Table 2**). The HADS-A score decreased from 9.27 ± 1.36 before surgery to 5.40 ± 1.89 after surgery, the HADS-D score decreased from 8.87 ± 1.38 before surgery to 4.70 ± 2.07 after surgery, the VAS score decreased from 69.23 ± 5.02 before surgery to 32.07 ± 9.98 after surgery, and the AOFAS score improved from 27.17 ± 8.57 before surgery to 65.47 ± 8.97 after surgery.

**Table 2:** Changes in evaluation indicators of patients in group A before surgery and at the final follow-up.

Evaluation indicators	VAS	AOFAS	HADS-A	HADS-D
preoperative	69.23 ± 5.02	27.17 ± 8.57	9.27 ± 1.36	8.87 ± 1.38
final follow-up	32.07 ± 9.98	65.47 ± 8.97	5.40 ± 1.89	4.70 ± 2.07
t value	20.505	-17.857	10.264	12.280
P value	P<0.001	P<0.001	P<0.001	P<0.001

At the final follow-up, patients in Group B showed significant improvements in all evaluation indicators compared to before surgery (P<0.001, **Table 3**). The HADS-A score decreased from 5.06 ± 1.10 before surgery to 2.75 ± 1.34 after surgery, the HADS-D score decreased from 4.94 ± 1.46 before surgery to 2.66 ± 1.12 after surgery, the VAS score decreased from 65.94 ± 5.40 before surgery to 23.87 ± 6.76 after surgery, and the AOFAS score improved from 37.22 ± 14.38 before surgery to 75.53 ± 9.28 after surgery.

**Table 3:** Changes in evaluation indicators of patients in group B before surgery and at the final follow-up.

Evaluation indicators	VAS	AOFAS	HADS-A	HADS-D
preoperative	65.94 ± 5.40	37.22 ± 14.38	5.06 ± 1.10	4.94 ± 1.46
final follow-up	23.87 ± 6.76	75.53 ± 9.28	2.75 ± 1.34	2.66 ± 1.12
t value	36.859	-17.744	13.554	11.025
P value	P<0.001	P<0.001	P<0.001	P<0.001

### Correlation analysis of psychological factors

Regarding the correlation between psychological factors and prognosis, this study found significant differences in postoperative scores between the two groups of patients, with Group A patients showing a poorer prognosis compared to Group B (P<0.001, **Table 4**). Group A patients had lower average AOFAS scores postoperatively compared to Group B patients, with higher average postoperative VAS, HADS-A, and HADS-D scores compared to Group B. Furthermore, we also found significant differences in the preoperative AOFAS and VAS scores between the two groups of patients (P<0.05, **Table 5**), but there were no significant differences in the degree of improvement in AOFAS and VAS scores after surgery between the two groups (P>0.05, **Table 6**).

**Table 4:** Analysis of the correlation between psychological factors and prognosis.

Evaluation indicators	VAS	AOFAS	HADS-A	HADS-D
t value	-3.759	4.336	-6.402	-4.785
P value	P<0.001	P<0.001	P<0.001	P<0.001

**Table 5:** Analysis of the correlation between psychological factors and preoperative VAS, AOFAS scores.

Evaluation indicators	VAS	AOFAS
groups t value	-2.486	3.367
P value	0.016	0.001

**Table 6:** Analysis of the correlation between psychological factors and the degree of improvement in VAS, AOFAS scores after surgery.

Evaluation indicators	VAS	AOFAS
groups t value	1.762	0.004
P value	0.084	0.997

**Figure 1:**
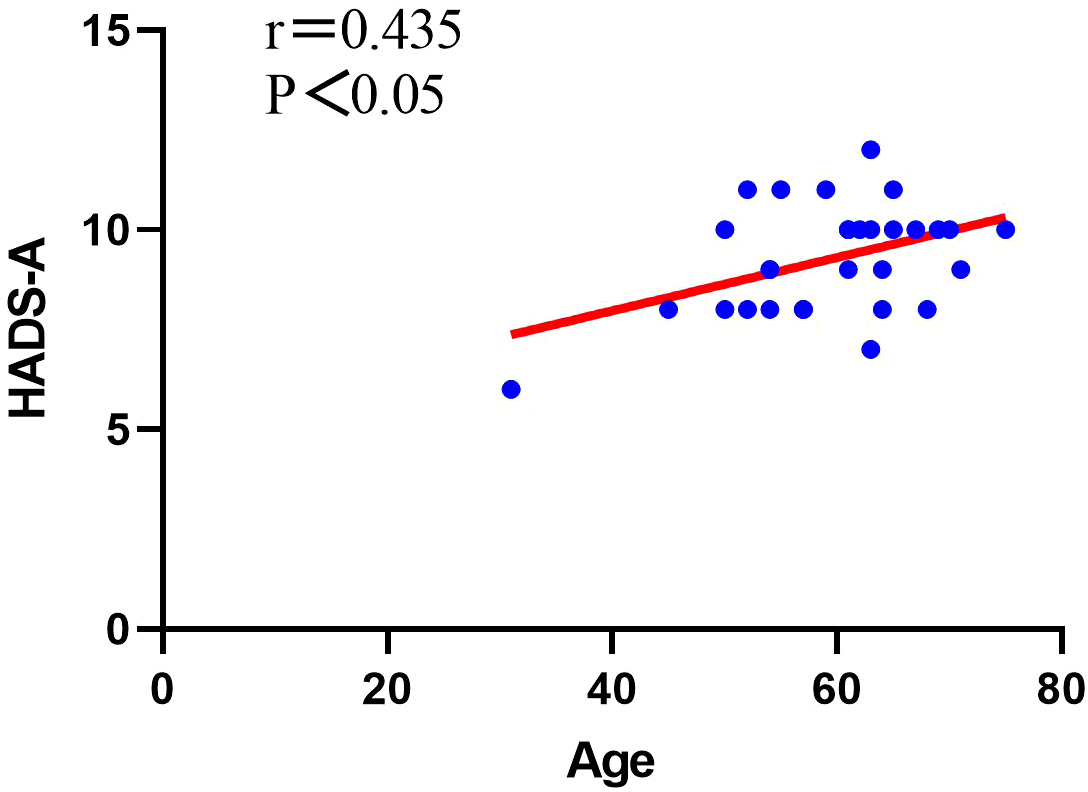
Analysis of the correlation between patient age and the level of preoperative anxiety.

**Figure 2:**
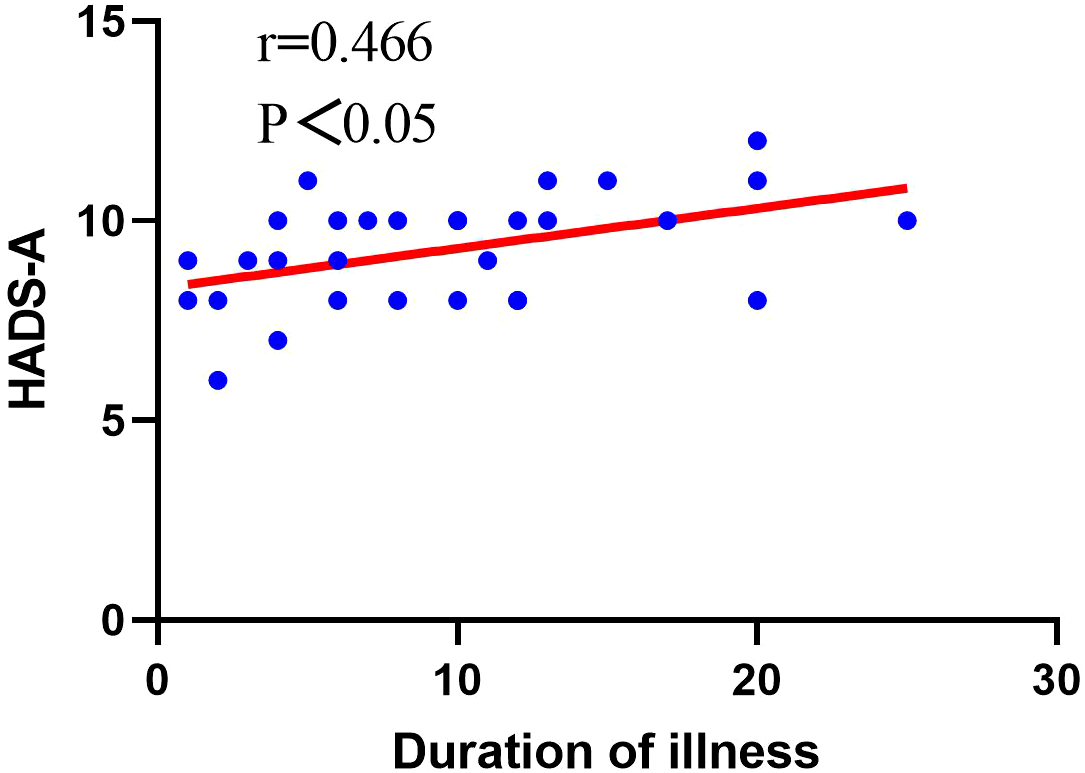
Analysis of the correlation between the duration of illness and the level of preoperative anxiety.

For Group A patients, we conducted a study on the correlation between patient gender, age, duration of illness, improvement in various evaluation indicators postoperatively, and psychological factors. Through our research, we found that in Group A patients, there was no correlation between gender and preoperative anxiety level (P>0.05, **Table 7**), but there was a significant correlation between gender and preoperative depression level (P<0.05, **Table 7**). There was no significant correlation between patient age and preoperative depression levels (P>0.05, **Table 7**), but there was a significant correlation between patient age and preoperative anxiety levels (P<0.05, **Table 7**), showing a positive correlation (**Figure 1**). There was no significant correlation between the duration of illness and preoperative depression levels (P>0.05, **Table 7**), but there was a significant correlation between the duration of illness and preoperative anxiety levels (P<0.05, **Table 7**), showing a positive correlation (**Figure 2**). Finally, we found that the level of preoperative anxiety/depression did not affect the degree of improvement in AOFAS and VAS scores after surgery (P>0.05, **Table 8**).

**Table 7:** Analysis of the correlation between gender, age, duration of illness, and preoperative psychological factors in Group A patients.

Evaluation indicators		HADS-A	HADS-D
Sex	t value	-0.059	-3.675
	P value	0.953	0.001
Age	r value	0.435	-0.161
	P value	0.016	0.395
Duration of illness	r value	0.466	0.039
	P value	0.010	0.838

**Table 8:** Analysis of the correlation between the degree of postoperative improvement and preoperative psychological factors in Group A patients.

Evaluation indicators		VAS	AOFAS
Anxiety	r value	0.190	0.268
	P value	0.316	0.152
depression	r value	-0.051	-0.044
	P value	0.788	0.817

## Discussion

Anxiety/depression disorders are negatively correlated with the clinical outcomes of orthopedic surgery (23–25). Cunningham et al. reported that preoperative depressive symptoms were associated with poorer postoperative clinical outcomes of TAA, with depressed patients showing smaller improvements in postoperative scores compared to non-depressed patients (26). Preoperative presence of anxiety/depression symptoms is a negative prognostic factor for total knee arthroplasty and total hip arthroplasty (27). Harmer et al. found that anxiety/depression disorders are common in patients undergoing primary total knee arthroplasty or total hip arthroplasty, and preoperative anxiety/depression disorders are associated with increased risks of postoperative infection, revision, and low patient satisfaction (28). However, there are currently few studies reporting the relationship between AA and anxiety/depression disorders. We retrospectively analyzed 62 patients with end-stage ankle OA undergoing AA treatment, and this study found a significant correlation between psychological factors and AA prognosis. Patients with anxiety/depression symptoms before surgery, regardless of pain, functional activity, or mental health, have worse recovery than patients without anxiety/depression symptoms before surgery, but there is still significant improvement compared to the preoperative clinical results. It is worth noting that while previous studies have investigated the relationship between psychological status and outcomes following various orthopedic procedures such as total knee arthroplasty, total hip arthroplasty, and total ankle arthroplasty, research specifically examining this relationship in ankle arthrodesis remains limited. Ankle arthrodesis and total ankle arthroplasty are fundamentally different procedures with distinct biomechanical outcomes and rehabilitation protocols, and findings from TAA studies cannot be directly extrapolated to AA patients. To our knowledge, this is among the first studies to specifically investigate the correlation between preoperative anxiety/depression and clinical outcomes following ankle arthrodesis for end-stage ankle osteoarthritis.

Goldberg et al. found that AA treatment for end-stage ankle OA can not only effectively improve patients’ pain and daily life activity ability, but also improve their social interaction ability to a certain extent (29). Shofer et al. reported that AA can significantly improve the activity level of patients with ankle OA (30). Tenenbaum et al. found that after AA surgery, patients’ gait speed, ankle joint torque, hip joint movement, and strength all significantly improved compared to before surgery (31). Our research results found that the VAS, AOFAS, and HADS scores of patients in both groups significantly improved after AA surgery compared to before surgery, and there was no significant difference in the degree of improvement in AOFAS and VAS scores after surgery between the two groups. During the follow-up process, we did not observe any complications such as infection, malunion, delayed healing, or nonunion in the patients. Therefore, we believe that AA can safely and effectively treat patients with end-stage ankle OA.

Compared with patients with hip OA and knee OA (28), patients with end-stage ankle OA have a higher incidence of anxiety/depression symptoms before surgery. Existing research reports that anxiety/depression disorders are related to a patient’s pain tolerance, pain sensitivity, and health-related quality of life (32, 33). Eisenach et al. found that there is a certain correlation between the degree of pain and depressive mood disorders, and the more severe the pain, the easier it is to cause depressive mood disorders (34). And there are research reports that patients with depression have higher pain sensitivity than patients without depression (35). James et al. found that the level of anxiety is related to pain tolerance, which may be related to high-anxiety patients having a higher focus on the surrounding environment and pain symptoms (36). Meanwhile, there are research reports that there is a significant positive correlation between the degree of pain and state anxiety (37). The results of this study show that patients with anxiety/depression symptoms also have more severe pain before surgery and a lower level of functional activity. We believe that the lower level of functional activity in patients is related to the more severe pain in patients with anxiety/depression.

In our study of Group A patients with poorer mental health, we found that there was a significant correlation between patient age and the level of preoperative anxiety, showing a positive correlation. This finding is consistent with clinical observations. Older patients facing surgery often express concerns about whether their body can withstand the procedure, fear of becoming a burden to their families, and uncertainty about returning to independent daily life after surgery, all of which may contribute to higher levels of preoperative anxiety. Furthermore, this study identified a significant correlation between patient sex and the level of preoperative depression. Currently, a large number of studies have reported on the correlation between gender and mental health. Van et al. conducted a study on 36,752 patients with depression across 23 countries and found that female patients reported higher levels of depression than male patients (38). The gender difference in depressive mood disorders may be related to socioeconomic status, family factors, and neurohormones, among other aspects (38–41). Our study also found a significant positive correlation between the duration of the patient’s illness and preoperative anxiety levels. Some studies have reported that the amount of physical activity correlates with the incidence of anxiety disorders (42–44). Patients with a longer duration of illness may have to reduce physical activity due to prolonged ankle joint pain, which can easily lead to the occurrence of anxiety disorders. Therefore, it is necessary to conduct psychological health assessments for elderly female patients with a longer duration of illness in clinical practice in order to develop personalized treatment plans for them. We also found no correlation between preoperative anxiety/depression levels and the degree of improvement in VAS and AOFAS scores postoperatively. Although the degree of improvement after surgery was the same for both groups, patients with preoperative anxiety/depression symptoms had a poorer prognosis because their overall condition before surgery was worse. Therefore, optimizing patients’ mental health preoperatively can effectively improve their prognosis. Notably, our study identified specific patient characteristics associated with preoperative psychological disturbances, including the significant correlation between female gender and depression levels, the positive correlation between patient age and anxiety levels, as well as the positive correlation between disease duration and anxiety levels. These findings provide clinically actionable insights that differ from previous studies and may help clinicians identify high-risk patients who would benefit from targeted preoperative psychological screening and intervention.

Our study has several limitations that should be acknowledged. First, as a retrospective observational study from a single tertiary orthopedic specialty hospital, it has inherent limitations including potential selection bias, recall bias, and limited generalizability to other populations. A large-sample, multicenter prospective randomized controlled trial would be needed to validate our findings. Second, the relatively short follow-up period may not capture long-term psychological changes or late complications, though our mid-term results still provide valuable insights. Third, the small sample size limited our statistical power, which may have prevented us from detecting some clinically meaningful associations that truly exist. Additionally, it restricted our ability to perform more sophisticated analyses such as multivariate regression to adequately control for potential confounding variables. Nevertheless, despite the limited sample size, our study still detected statistically significant differences between the two groups, which may suggest a relatively strong association between anxiety/depression and surgical outcomes. Fourth, several important moderator variables were not assessed in our study, which could potentially influence the relationship between psychological status and surgical outcomes. These include social support systems, socioeconomic factors, pain coping strategies, rehabilitation compliance, body mass index, and patient expectations. Future studies should incorporate these moderator variables using multivariate regression analysis to better understand their role in surgical outcomes. Additionally, we relied solely on the HADS for psychological assessment. While validated, using multiple psychological assessment tools could provide a more comprehensive evaluation of psychological status. Despite these limitations, to our knowledge, this is the first study to specifically examine the correlation between psychological factors and ankle arthrodesis outcomes, providing important preliminary evidence for this relationship.

## Conclusion

Patients with end-stage ankle OA are more likely to experience anxiety/depression symptoms preoperatively, and those with these symptoms have poorer functional activity levels and more severe pain before surgery. AA can safely and effectively improve pain, functional activity, and psychological status in patients with end-stage ankle OA, and there is a significant correlation between preoperative anxiety/depression symptoms and the prognosis of AA.

## Data Availability

The datasets generated and/or analyzed during the current study are not publicly available due to patient privacy and confidentiality requirements, but are available from the corresponding author upon reasonable request and with appropriate institutional approval. The data that support the findings of this study are available from the corresponding author.
